# Supplementary figures and images for: Single-Cell Molecular Characterization to Partition the Human Glioblastoma Tumor Microenvironment Genetic Background
Source: Cells. 2022 Mar 26;11(7):1127. doi: 10.3390/cells11071127 (PMC8998055; doi:10.3390/cells11071127)

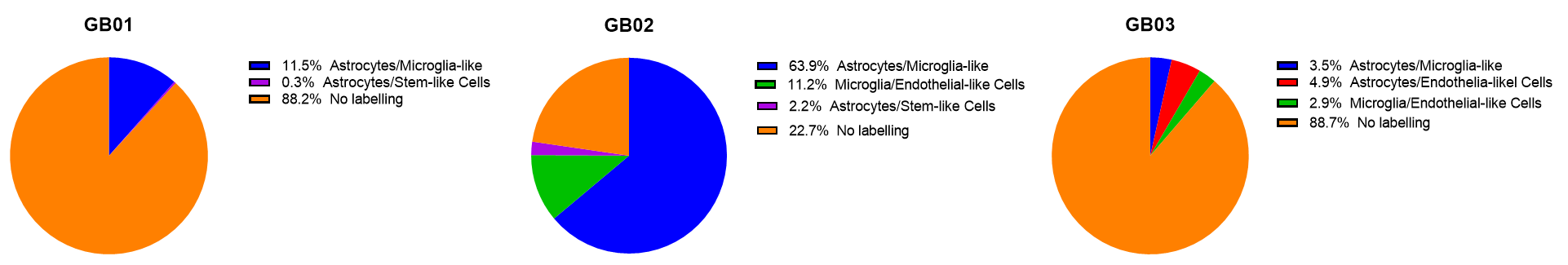

Supplement: Supplementary file 1 [file cells-11-01127-s001.zip › Figure S1.png]
